# Fructose transport-deficient *Staphylococcus aureus* reveals important role of epithelial glucose transporters in limiting sugar-driven bacterial growth in airway surface liquid

**DOI:** 10.1007/s00018-014-1635-y

**Published:** 2014-05-09

**Authors:** James P. Garnett, Daniela Braun, Alex J. McCarthy, Matthew R. Farrant, Emma H. Baker, Jodi A. Lindsay, Deborah L. Baines

**Affiliations:** Institute for Infection and Immunity, St George’s, University of London, Tooting, London, SW17 0RE UK

**Keywords:** Glucose, Glucose transporters (GLUTs), Hyperglycaemia, Airway epithelium, *Staphylococcus aureus*, Respiratory infection

## Abstract

Hyperglycaemia as a result of diabetes mellitus or acute illness is associated with increased susceptibility to respiratory infection with *Staphylococcus aureus*. Hyperglycaemia increases the concentration of glucose in airway surface liquid (ASL) and promotes the growth of *S. aureus* in vitro and in vivo. Whether elevation of other sugars in the blood, such as fructose, also results in increased concentrations in ASL is unknown and whether sugars in ASL are directly utilised by *S. aureus* for growth has not been investigated. We obtained mutant *S. aureus* JE2 strains with transposon disrupted sugar transport genes. NE768(*fruA*) exhibited restricted growth in 10 mM fructose. In H441 airway epithelial-bacterial co-culture, elevation of basolateral sugar concentration (5–20 mM) increased the apical growth of JE2. However, sugar-induced growth of NE768(*fruA*) was significantly less when basolateral fructose rather than glucose was elevated. This is the first experimental evidence to show that *S. aureus* directly utilises sugars present in the ASL for growth. Interestingly, JE2 growth was promoted less by glucose than fructose. Net transepithelial flux of d-glucose was lower than d-fructose. However, uptake of d-glucose was higher than d-fructose across both apical and basolateral membranes consistent with the presence of GLUT1/10 in the airway epithelium. Therefore, we propose that the preferential uptake of glucose (compared to fructose) limits its accumulation in ASL. Pre-treatment with metformin increased transepithelial resistance and reduced the sugar-dependent growth of *S. aureus*. Thus, epithelial paracellular permeability and glucose transport mechanisms are vital to maintain low glucose concentration in ASL and limit bacterial nutrient sources as a defence against infection.

**Electronic supplementary material:**

The online version of this article (doi:10.1007/s00018-014-1635-y) contains supplementary material, which is available to authorized users.

## Introduction

Increased risk of *Staphylococcus aureus* infection in the respiratory tract is associated with hyperglycaemia [1, 2]. Diabetes is a risk factor for nasal colonisation with *S. aureus* [3], increased pathogen load in cystic fibrosis (CF) [4, 5] and increased exacerbation frequency in people with chronic obstructive pulmonary disease (COPD) [1, 6].In an intensive care unit, patients with hyperglycaemia had more *S. aureus* in sputum and this was associated with increased glucose concentration in the thin layer of fluid that lines the airways (airway surface liquid, ASL) [2].

Glucose concentration in human ASL is normally much lower than that of blood at ~0.4 mM, [7–9]. However, ASL glucose concentrations are elevated in people with respiratory disease including acute viral rhinitis [10], COPD [1] and CF [9]. ASL glucose concentrations are also increased in experimental [11] and diabetic hyperglycaemia [10] and are further increased in people with both respiratory disease and diabetes [5, 9].

We developed an in vitro model of airway glucose homeostasis and showed that, under normal conditions, glucose predominantly diffuses from blood/interstitial fluid across the respiratory epithelium into the ASL via paracellular pathways, and this is limited by epithelial permeability [8, 12–15]. Uptake via apical and basolateral GLUT transporters also restricts glucose accumulation in ASL [12–16] and rapid metabolism of glucose helps to maintain low intracellular glucose concentrations. This provides a driving force for glucose uptake and limits the transcellular transport of glucose, leading to equilibrated ASL and intracellular glucose concentrations [13, 14]. In this model, increasing the diffusion gradient for glucose across the epithelium (e.g. hyperglycaemia) and increasing paracellular diffusion of glucose via reduced transepithelial resistance (*R*_T_) (e.g. inflammation) result in elevated ASL glucose concentration [12, 13, 17].

We have shown that *S. aureus* growth is promoted by glucose in microbial culture [5] and that basolateral hyperglycaemia promotes apical growth of *S. aureus* in airway epithelial co-culture [12, 18]. *S. aureus* can also utilise other sugars such as fructose. This sugar has recently received much attention, as it is included as a sweetener in many drinks. In a study of 39 clinical samples, plasma fructose levels were lower than glucose, 46 ± 25 µM compared to 6.19 ± 2.72 mM, but can reach 300 µM particularly after ingestion of high-fructose corn syrup [19, 20]. A high-fructose diet has been shown to lead to insulin insensitivity and accelerate the development of type II diabetes in rats [21–23]. Elevation of systemic fructose could therefore contribute to the hyperglycaemia-induced growth of *S. aureus* in the airways. It is not known if fructose crosses the lung epithelial cell membrane into ASL where it could provide an additional growth substrate for *S. aureus*. Moreover, there is also no evidence that sugars present in ASL (e.g. glucose or fructose) are directly utilised by *S. aureus* for growth.

We therefore investigated how glucose and fructose modified growth of *S. aureus* carbohydrate transporter mutants in microbial and airway epithelial co-culture. Our data indicate that fructose, in addition to glucose, crosses the airway epithelial barrier and that sugars in ASL are utilised by *S. aureus* to promote growth. In addition, our data indicate that transport of glucose and fructose by airway epithelial cells decreased the transepithelial flux of these sugars. The preferential uptake of glucose over fructose also sheds light on the identity of GLUT transporters involved.

## Methods

### Bacterial culture

*S. aureus* JE2 is a USA300 community-associated methicillin-resistant *S. aureus* used as the parent in construction of the Nebraska sequence-defined transposon insertion library [24]. JE2 and five transposon library mutants with insertions in predicted glucose or fructose sugar transport pathway genes were obtained from the network on antimicrobial resistance in *Staphylococcus aureus* (NARSA) and are listed in Table 1. For microbial culture, JE2 strains were inoculated into brain heart infusion broth (BHI) and grown overnight at 37 °C. The following day, the culture was diluted to an OD_540_ of 0.05 using RPMI. Then, 100 µl of this adapted culture was added into either 20 ml of glucose-free RPMI, RPMI + 10 mM glucose or RPMI + 10 mM fructose. Cultures were incubated in a shaking water bath at 37 °C and 80 rpm for 24 h. OD measurements were taken every hour up to 8 h then again at 24 h. Samples were also removed for Miles and Misra quantification of colony forming units (CFU) on BHI agar. Plates were incubated overnight at 37 °C and CFU counted the following day.

**Table 1:** *S. aureus* parent and mutant strains from the Nebraska sequence-defined transposon library [22]

Strain name	Transposon insertion site	Gene description	Accession number
JE2	No mutation	Parent strain	
NE39(*ptsG*)	*ptsG*	Phosphotransferase system, glucose-specific II ABC component	SAUSA 300_247C
NE768	*fruA*	Fructose-specific permease	SAUSA 300_0685
NE172	*ptsG*	Phosphotransferase system, glucose-specific II BC component domain protein	SAUSA 300_0191
NE1944	*crr*	Phosphotransferase system, glucose-specific II A component	SAUSA 300_1315
NE1405	*gluC*	Probable glucose uptake protein	SAUSA 300_2210

For airway epithelial co-culture, a single colony of *S. aureus* strain JE2 or mutant was incubated overnight at 37 °C in RPMI media containing glucose or fructose. The following day, fresh media was inoculated with 200 μl of the overnight culture and grown at 37 °C to log phase, OD_540_ of 0.5 (approximately 2 × 10^7^ CFU/ml).

### H441 airway epithelial cell culture and in vitro co-culture model

H441 epithelial cells were grown on permeable membrane supports (Transwells, Corning, MA, USA) at air–liquid interface to form lung epithelial cell layers, as previously described [12]. Monolayers were pre-treated with vehicle or 1 mM metformin (added to the basolateral medium) 18 h prior to apical inoculation with *S. aureus*. Monolayer transepithelial resistance (*R*_T_) was measured using an epithelial volt ohmmeter (EVOM; World Precision Instruments, UK). For resistance measurements and bacterial co-cultures, the basolateral side of H441 monolayers was bathed in Krebs salt solution (in mM): NaCl, 117; NaHCO_3_, 25; KCl, 4.7; MgSO_4_, 1.2; KH_2_PO_4_, 1.2; CaCl_2_, 2.5 (equilibrated with 5 % CO_2_ to pH 7.3–7.4), containing 5 mM glucose or 20 mM glucose or 5 mM glucose + 15 mM fructose.

*S. aureus* cultures were diluted in glucose-free RPMI media and approximately 5 × 10^5^ CFU of bacteria in 50 μl was applied to the apical surface of H441 monolayers. H441-*S. aureus* co-cultures were placed in a CO_2_ incubator at 37 °C for 7 h, after which each was homogenised and CFU calculated by plating out serial dilutions.

### Paracellular flux and uptake experiments

Paracellular movement of glucose and fructose across H441 monolayers was measured by analysis of radiolabelled [^14^C]-l-glucose, [^14^C]-d-glucose and [^14^C]-d-fructose transepithelial flux. Experiments were initiated by adding 0.5 ml of Krebs salt solution containing 1.0 μCi of radiolabeled sugar plus non-radiolabelled d-glucose (5 mM) and d-fructose (5 mM) to the basolateral side of the transwells and 0.1 ml of sugar-free Krebs solution to the apical side. Apical and basolateral samples were taken after 1 h and the concentration of radiolabelled glucose was analysed using a scintillation counter. For [^14^C]-d-glucose and [^14^C]-d-fructose experiments, paracellular flux was calculated by compensating for total monolayer uptake.

Uptake experiments on H441 monolayers were initiated by replacing the medium with 0.5 ml transport medium containing 1.0 μCi of [^14^C]-d-glucose or [^14^C]-d-fructose plus 5 mM of non-radiolabelled equivalent d-glucose and d-fructose, to either the basolateral or apical side of the monolayer, followed by incubation at room temperature for 10 min. Preliminary experiments indicated that the uptake was linear between 0 and 10 min (data not shown). Uptake was terminated by adding 2 ml ice-cold stop solution (15 mM HEPES buffer (pH 7.6); 135 mM choline Cl; 5 mM KCl; 0.8 mM MgSO_4_; 1.8 mM CaCl_2_ and 0.2 mM HgCl_2_). The cells were then rinsed twice with 2 ml stop solution and lysed in 0.5 ml of 10 mM Tris–HCl (pH 8.0) with 0.2 % SDS. Lysed samples were added to 2 ml scintillation cocktail and radio-active emissions determined using a scintillation counter to quantify glucose and fructose uptake.

## Chemicals and reagents

All chemicals and reagents were obtained from Sigma, Poole, UK unless otherwise stated.

### Statistical analysis

Values are reported as mean ± SEM. Statistical analysis was performed using analysis of variance (ANOVA) and post hoc Bonferroni multiple comparison or Student’s *t* test. *p* values of <0.05 were considered significant.

## Results

### Growth of *S. aureus* mutants in glucose and fructose

The growth curves of parent strain JE2 and the 5 isogenic mutants NE39(*ptsG*), NE768(*fruA*), NE1944(*crr*), NE172(*ptsG*) and NE1405(*gluC*) were characterised over 24 h. To identify genes important for *S. aureus* sugar uptake and growth, the growth of all isogenic mutants was compared to JE2 under three conditions: RPMI medium, RPMI supplemented with 10 mM glucose and RPMI supplemented with 10 mM fructose.

The Log_10_OD_540_ indicated that the growth of all six strains in RPMI in the absence of sugar was heavily restricted up to 8 h (Supplemental Figure 1). There was an observed increase in the Log_10_OD_540_ at 24 h of all strains. Importantly, there was no striking difference in the growth of each isogenic strain compared to JE2 parent strain, indicating that gene disruptions did not impact growth in the absence of sugar.

In the presence of 10 mM glucose, the Log_10_OD_540_ for all strains was increased during lag phase and isolates began log phase by 6 h (Supplemental Figure 1). Strain NE39(*ptsG*) had the same growth characteristics as the parent strain JE2 but strains NE768(*fruA*), NE1944(*crr*), NE172(*ptsG*) and NE1405(*gluC*)were observed to grow more slowly than the parent strain JE2 between 7 and 8 h. After 24 h, all 6 strains reached a similar Log_10_OD_540_, which was higher than in RPMI alone (Supplemental Figure 1).

In the presence of 10 mM fructose, the early Log_10_OD_540_ for all strains was lower than in glucose. All strains except NE768(*fruA*) began log phase by 6 h(Supplemental Figure 1). NE768(*fruA*) growth was noticeably less than the JE2 parent strain after 7 and 8 h (*n* = 2 each strain). After 24 h, all 6 strains reached a similar Log_10_OD_540_, which was higher than in RPMI alone (0.276 ± 0.002 compared to 0.177 ± 0.005, respectively, *p* < 0.0001, *n* = 6).

**Fig. 1:**
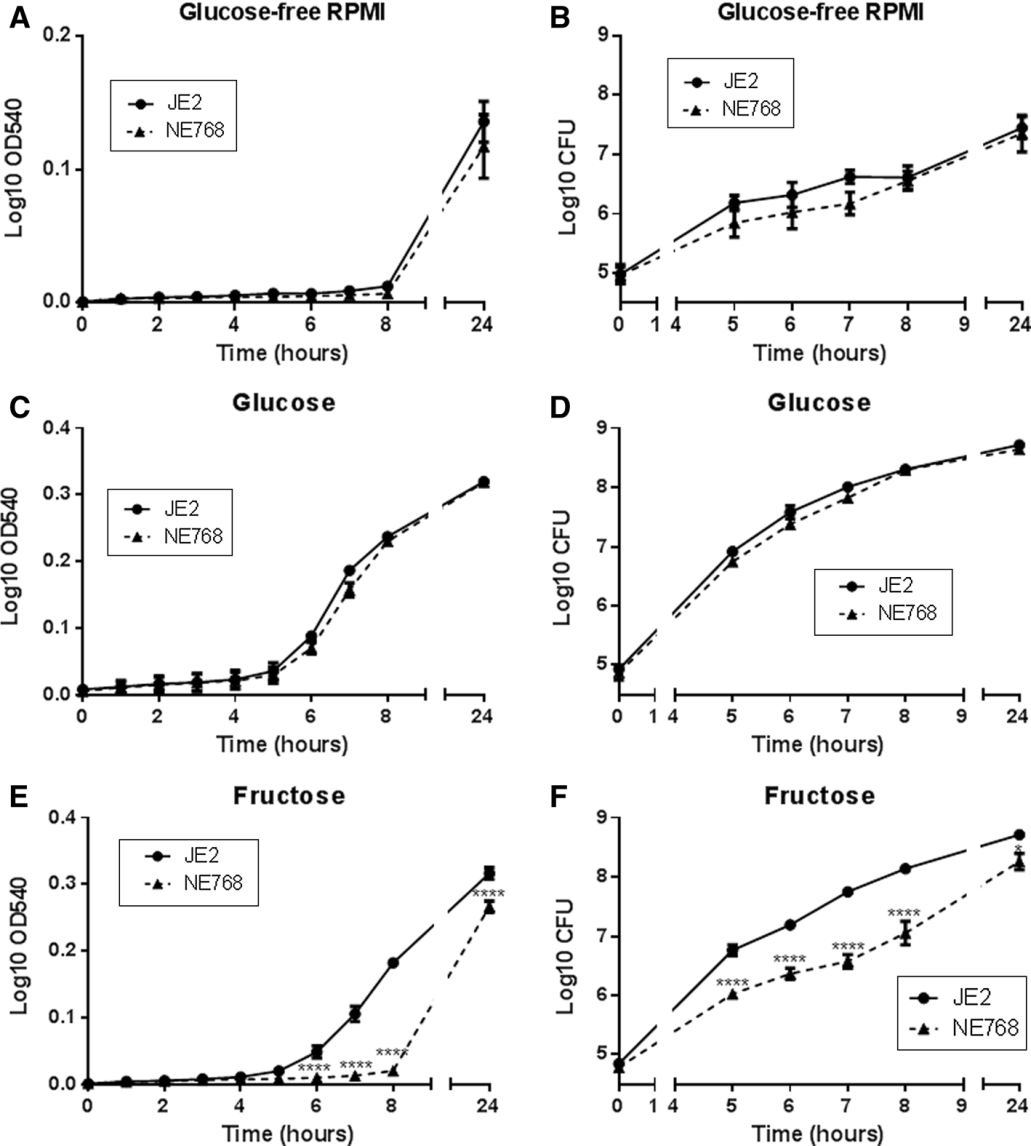
Growth of mutant *S. aureus* strains in the presence of glucose and fructose. Growth of *S. aureus* parent strain (JE2) and mutant strains NE768(fruA), NE172(ptsG), NE1944(crr) measured as OD540 or viable cell assay (CFU/ml) over 24 h, in glucose-free RPMI (**a**, **b**), RPMI supplemented with 10 mM glucose (**c**, **d**) or RPMI supplemented with 10 mM fructose (**e**, **f**), *n* = 5. *p < 0.05, ****p < 0.0001, compared to JE2

From these first observational experiments, we then focussed our studies on NE768 (*fruA* fructose transporter mutant) and the parent strain JE2.

The growth of JE2 was similar in both 10 mM glucose and fructose (*p* > 0.05, *n* = 5, Fig. 1c–f). NE768(*fruA*) exhibited similar growth to JE2 in 10 mM glucose (*p* > 0.05, *n* = 5, Fig. 1c, d). However, the growth of NE768(*fruA*) was significantly inhibited compared to JE2 when grown in 10 mM fructose, Log_10_OD_540_ at 8 h was 0.014 ± 0.006 and 0.181 ± 0.02, respectively (*p* < 0.0001, *n* = 5, Fig. 1e).Viable counts confirmed 10 mM glucose-induced similar growth of JE2 and NE768(*fruA*) (*p* > 0.05, *n* = 5, Fig. 1d), while viable counts of JE2 in fructose at 8 h were significantly higher than NE768(*fruA*) (Log_10_ CFU was 8.14 ± 0.06 and 7.05 ± 0.20, respectively; *p* < 0.0001, *n* = 5, Fig. 1f). These data confirmed that mutation of the *fruA* gene impacted the ability of strain NE768(*fruA*) to utilise fructose.

### Effect of sugar on transepithelial resistance (*R*_T_)

**Fig. 2:**
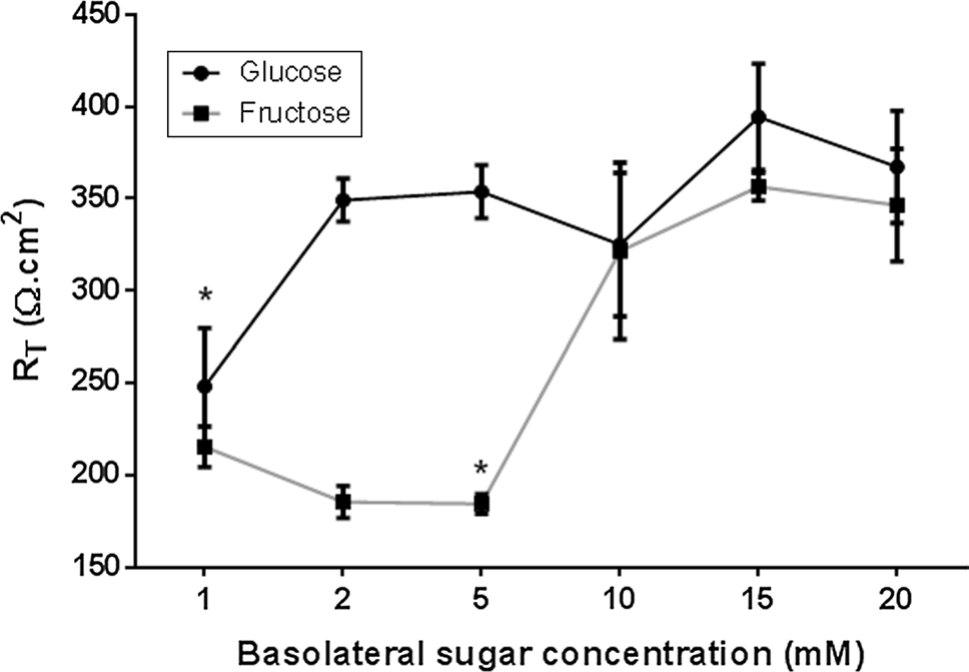
Sugar-induced changes in transepithelial electrical resistance across airway monolayers. Transepithelial resistance (*R*_T_) across H441 monolayers after 24 h incubation with basolateral Krebs salt solution supplemented with 1, 2, 5, 10, 15, 20 mM glucose or fructose, *n* = 5. *p < 0.05 compared to 2 mM glucose, 5 mM glucose, 15 mM glucose, 20 mM glucose, 15 mM fructose and 20 mM fructose

In our co-culture model, we firstly determined whether changing the glucose or fructose concentration of basolateral solution bathing the H441 airway epithelial cells could adversely affect transepithelial electrical resistance (*R*_T_), as we previously found that high concentrations of glucose impaired Calu-3 airway epithelial monolayer resistance [15]. Supplementation with 2–20 mM glucose had no significant effect on *R*_T_ in H441 cells grown at air–liquid interface over a time period of 7 h (*p* > 0.05, *n* = 5, Fig. 2). However, H441 *R*_T_ was only stable with high concentrations of fructose (10–20 mM). *R*_T_ declined significantly when the glucose concentration was reduced to 1 mM (*p* < 0.05, *n* = 5), or fructose was lowered to 5 mM (*p* < 0.05, *n* = 5). To avoid sugar-induced changes in *R*_T_ during H441-*S. aureus* co-culture experiments, the basolateral solution contained 5 mM glucose(control), which was then supplemented with 15 mM glucose or fructose to measure sugar-induced bacterial growth.

### Effect of elevation of basolateral glucose or fructose concentration on the apical growth of JE2 or NE768(fruA) in epithelial-bacterial co-culture

**Fig. 3:**
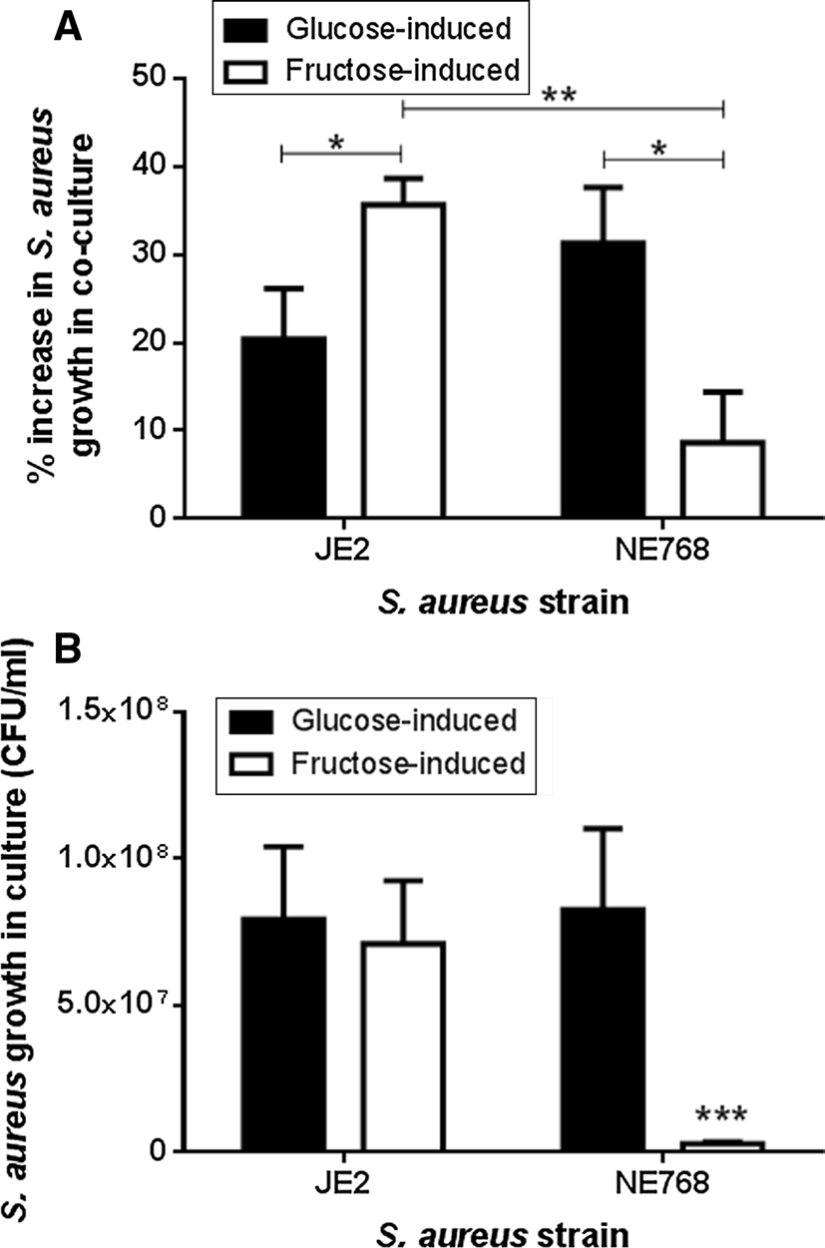
Apical *S. aureus* growth on airway epithelial monolayers is dependent on basolateral sugar concentration. **a** H441 airway epithelia-bacteria co-cultures were grown in basolateral Krebs salt solution supplemented with 5 mM glucose (control), 20 mM glucose (glucose-induced growth) or 5 mM glucose + 15 mM fructose (fructose-induced growth). *S. aureus* JE2 and NE768(*fruA*)CFU were measured 7 h post infection (% increase compared to growth in the presence of 5 mM basolateral glucose), *n* = 5. *p < 0.05, **p < 0.01. **b** Increase in the number of JE2 and NE768(*fruA*) *S. aureus* (CFU/ml) in microbial culture over 7 h in the presence of 10 mM glucose or 10 mM fructose, compared to glucose-free control, *n* = 10. *p < 0.05 compared to all other *bars*

Increasing the basolateral glucose concentration from 5 to 20 mM increased the growth of JE2 and NE768(*fruA*) on the apical surface of H441 airway epithelial monolayers after 7 h (*p* < 0.05, *n* = 5, Fig. 3a). Addition of 15 mM fructose (plus 5 mM glucose) to the basolateral chamber also increased the apical growth of JE2 (*p* < 0.05, *n* = 5). However, the fructose-induced growth of NE768(*fruA*)was significantly less than that of JE2 (*p* < 0.01, *n* = 5). These data indicate that *S. aureus* directly utilises sugars in the ASL for growth. Analysis of sugar-induced growth in the epithelial-bacterial co-cultures also indicated that fructose induced more growth of JE2 than glucose (*p* < 0.05, *n* = 5, Fig. 3a). This was in contrast to JE2 growth in the presence of these two sugars in microbial culture (*p* > 0.05, *n* = 10, Fig. 3b).

### Fructose and glucose uptake and transepithelial flux across airway epithelial monolayers

**Fig. 4:**
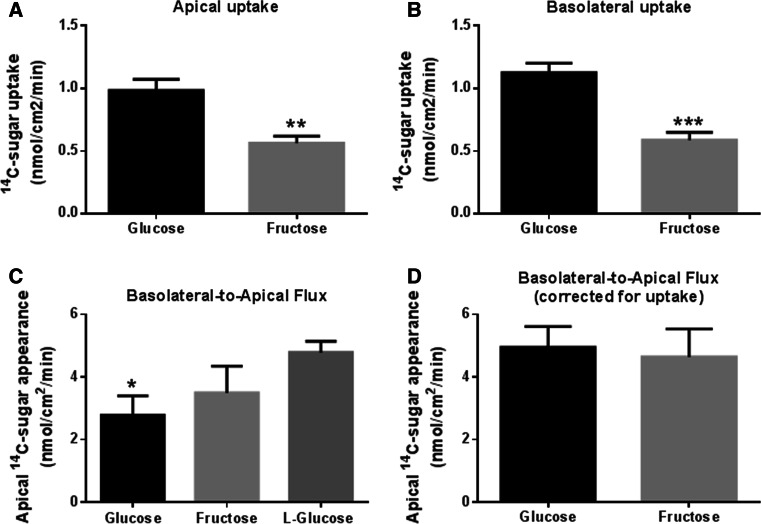
Transepithelial flux and uptake of glucose and fructose across airway epithelial monolayers. **a** Uptake of ^14^C-d-glucose and ^14^C-D-fructose across the apical membrane of H441 monolayers, *n* = 8. **b** Uptake of ^14^C-d-glucose and ^14^C-d-fructose across the basolateral membrane of H441 monolayers, *n* = 8. **c** Basolateral-to-apical transepithelial flux of d-glucose, d-fructose and l-glucose across H441 monolayers, measured by adding radiolabelled sugar to the basolateral surface and monitoring its appearance of the apical surface over 1 h, *n* = 4. **d** Calculated basolateral-to-apical transepithelial flux of d-glucose and d-fructose across H441 monolayers, after compensation for epithelial uptake, *n* = 4. *p < 0.05, **p < 0.01, ***p < 0.001

A comparison of d-fructose and d-glucose uptake by H441 monolayers revealed that fructose uptake was significantly less than glucose across both apical and basolateral membranes (*p* < 0.01 and *p* < 0.001, respectively; *n* = 8, Fig. 4a, b).

The rate of basolateral-to-apical net transepithelial flux of d-glucose was lower than that of l-glucose (not transported or metabolised) (*p* < 0.01, *n* = 4, Fig. 4c) and lower than that of d-fructose (*p* < 0.05, *n* = 4). Given that net flux of sugars = transepithelial flux − uptake, we calculated transepithelial flux for glucose and fructose. We found that calculated transepithelial flux was similar for glucose and fructose indicating that both similarly cross the epithelium (*p* > 0.05, *n* = 4, Fig. 4d). Moreover, these values were also similar to that of l-glucose which is not transported or metabolised (Fig. 4c, d). Thus, the difference in the rate of apical appearance of d-fructose and d-glucose could be accounted for by the difference in uptake of the two sugars.

### Effect of metformin on fructose-induced bacterial growth

**Fig. 5:**
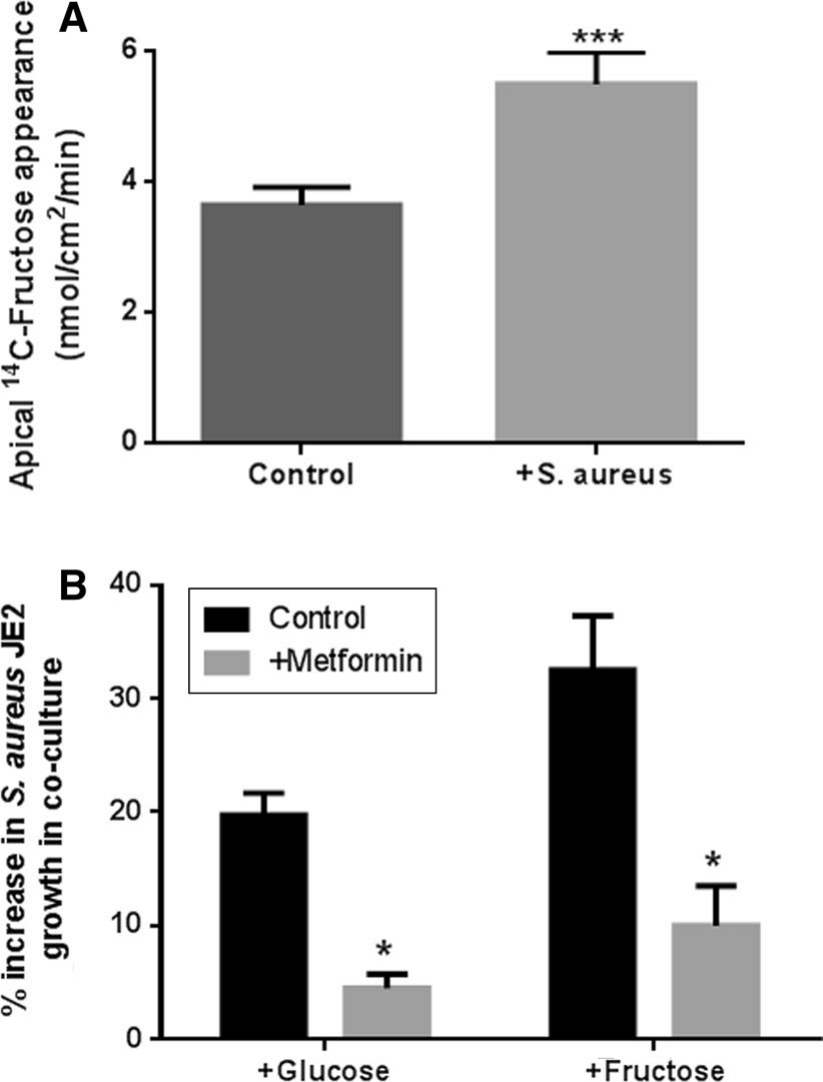
Metformin reduced the transepithelial flux of fructose and inhibited fructose-induced *S. aureus* JE2 growth across airway epithelial monolayers. **a** The effect of metformin pre-treatment of H441 airway epithelial monolayers (1 mM; 18 h pre-treatment) on the fructose-induced growth of apical *S. aureus* JE2. H441 airway epithelia-bacteria co-cultures were grown in basolateral Krebs salt solution supplemented with 5 mM glucose (control) or 5 mM glucose + 15 mM fructose (fructose-induced growth). *S. aureus* CFU were measured 7 h post infection (% increase compared to growth in the presence of 5 mM basolateral glucose), *n* = 4. **b** Transepithelial flux of fructose across untreated and metformin pre-treated H441 monolayers, measured by adding radiolabelled d-fructose to the basolateral surface and monitoring its appearance of the apical surface, *n* = 4. *p < 0.05, ***p < 0.001, compared to control

We previously showed that apical addition of *S. aureus* decreased transepithelial resistance and increased paracellular permeability to glucose [18]. In these experiments, apical addition of *S. aureus* also significantly increased the rate of basolateral-to-apical flux of d-fructose (*p* < 0.01, *n* = 4, Fig. 5a). Moreover, we previously found that pre-treatment of H441 airway epithelial monolayers cells with 1 mM metformin attenuated *S. aureus*-induced changes in the paracellular flux of glucose and reduced glucose-induced apical *S. aureus* growth. In the current study, metformin pre-treatment of the epithelium also reduced fructose-induced *S. aureus* growth, consistent with paracellular movement of fructose into the ASL providing a stimulus for bacterial growth (*p* < 0.05, *n* = 4, Fig. 5b). In support of this concept, it should be noted that metformin was removed and the epithelial cells were washed prior to the addition of bacteria in co-culture experiments. Furthermore, metformin had no direct effect on *S. aureus* growth in microbial culture (*n* = 3, Supplemental Figure 2).

## Discussion

We previously showed that elevation of basolateral glucose promoted the growth of apical/luminal *S. aureus* in an in vitro model of human airway and that hyperglycaemia promoted the growth of *S. aureus* in mouse airway [18].We showed that more glucose crossed the epithelial barrier into ASL under these conditions and we proposed that this provided a nutrient source for *S. aureus* [18].

Glucose and other sugars are growth substrates for respiratory pathogens such as *S. aureus* [25–27]. However, glucose may alter other factors in ASL such as the secretion of antimicrobial proteins [28]. Glucose is also a known trigger for regulatory pathways in *S. aureus*, altering metabolic pathways, expression of virulence factors, and biofilm production [29, 30].

In this study, we wanted to show that sugars present in ASL were taken up and utilised by *S. aureus* for growth. To do this, we used five previously uncharacterized isogenic mutant strains of *S. aureus* JE2. A number of these strains contained disruptions of the putative phosphotransferase system (PTS) which catalyses the transport and phosphorylation of monosaccharides, disaccharides and other sugar derivatives in bacteria. The system is complex and consists of three functional units, IIA, IIB and IIC to which a number of microbial genes contribute. The *crr* gene is the structural gene for III^Glc^, a IIA component of the PTS, and the *ptsG* operon encodes glucose (IICBA) and glucoside-specific enzymes that are sugar permeases, delivering specific sugars to the PTS [31]. In *E. coli crr* mutants, only 2–3 % in vitro phosphorylation of methyl α-glucoside activity was retained [32]. Thus, we expected that disruption of these genes would have a potent effect on glucose uptake and growth in *S. aureus*. Instead, glucose promoted the growth of all mutants and growth of NE1944(*crr*) and NE172(*ptsG*) was only inhibited by ~10 and 20 %, respectively, at 8 h. Our findings indicate that either transposon insertion did not lead to complete loss of function of *crr* and *ptsG* genes in *S. aureus* or, more likely, that other pathways provided routes for glucose uptake and metabolism in these mutants [33]. It is possible that the mannose PTS, EIIABCD^Man^, which has wide substrate specificity and can transport glucose, could provide such a substitute transport system [34–36]. Alternatively, an independent glucose uptake pathway may compensate for the mutations. NE1405(*gluC*) was modified in its glucose uptake protein *glcU* which may be independent of the PTS pathway. This mutant also grew well in the presence of glucose, presumably because it is compensated by an intact PTS glucose uptake system.

NE768(*fruA*) carried a mutation in the *fruA* gene which encodes the putative fructose-specific permease that interacts with the PTS. Fructose promoted the growth of all mutants tested, except NE768(*fruA*). Growth of this mutant was significantly suppressed for up to 8 h but reached similar levels to all other mutants at 24 h in microbial culture. These data indicate that *fruA* disruption led to an inability to efficiently utilise fructose for growth.

Because the glucose transporter mutations had little impact on bacterial growth, we used NE768(*fruA*) to identify whether fructose could cross the epithelial barrier and promote apical *S. aureus* growth. We found that elevation of basolateral fructose or glucose concentration promoted the apical growth of JE2 but only glucose effectively promoted the growth of NE768(*fruA*). These findings indicate that fructose also diffuses across the epithelial barrier into ASL and key to our hypothesis, the growth of *S. aureus* was dependent on the ability of the bacteria to take up and metabolise fructose. This is the first experimental evidence to show that *S.aureus* directly utilises sugars present in the ASL.

We noted that apical growth of JE2 was greater when the concentration of basolateral fructose, rather than glucose, was raised in epithelial-bacterial co-culture. This was in contrast to bacterial culture, where there was no difference in fructose- or glucose-induced growth over a similar time period. We previously showed that glucose diffuses across the airway epithelium into ASL, but its concentration is kept low by epithelial glucose transport and metabolism [12, 13, 16, 37]. Our data show that both glucose and fructose similarly diffused from the basolateral compartment into ASL and that paracellular flux was increased by the presence of bacteria [18]. However, the basolateral and apical uptake of fructose by H441 cells was significantly lower than that of glucose. Since pharmacological inhibition of GLUT-mediated glucose uptake with phloretin led to increased apical accumulation of glucose [13], we propose that, in this experimental model, reduced fructose uptake resulted in increased accumulation of fructose in ASL providing more availability of nutrients for wild-type JE2 growth.

These data and our finding that the *R*_T_ of epithelial monolayers decreased in fructose concentrations lower than 10 mM, but only in glucose concentrations lower than 2 mM, also shed important light on the potential identity of glucose transporters (GLUTs) that regulate glucose uptake and ASL glucose concentration. We identified glucose transporters GLUT2 and GLUT10 in H441 cells [15]. GLUT1 and GLUT10 were identified in primary human bronchiolar epithelial cells (HBE) [14]. GLUT2 transports glucose and fructose with similar low affinity (*K*_m_ ~ 15 mM) [38, 39]. GLUT1 and GLUT10 transport glucose with higher affinity (*K*_m_ ~ 5.0 and 0.3 mM, respectively), but do not transport fructose [40, 41]. Thus, the increased uptake of glucose compared to fructose points towards the involvement of GLUT1 and/or GLUT10 in this process. In our previous studies, we were unable to identify GLUT1 in H441 cells, but GLUT10 protein expression correlated with increased glucose uptake in response to pro-inflammatory agents [12]. As GLUT10 is localised to the mitochondria in vascular smooth muscle cells [42, 43] and mutations of the SLC2A10 gene are associated with arterial tortuosity syndrome [44], more work is now required to elucidate this potentially distinct role of GLUT10 in the airway.

Treatment of H441 cells with metformin inhibited both glucose-and fructose-induced growth of JE2 and was associated with restoration of airway epithelial *R*_T_ consistent with our previous findings [18]. Both glucose and fructose are similar sized, non-charged, hexose molecules. Thus, these data further support our model where by metformin reduced paracellular flux of sugars limiting their accumulation in ASL and availability as nutrients for *S. aureus* growth [13].

In conclusion, we characterised a number of *S. aureus* carbohydrate transporter mutants and identified NE768(*fruA*) (a *fruA* mutant) with a reduced ability to utilise fructose as a growth substrate. Use of this mutant in epithelial-bacterial co-culture showed that *S. aureus* directly utilised sugars present in ASL for growth. Both fructose and glucose diffuse across the airway epithelium into ASL. However, epithelial uptake of fructose is less than that of glucose. We propose this is because key transporters present in the airway epithelium preferentially transport glucose (e.g. GLUT1/10). This decreases the diffusion gradient for paracellular movement of glucose compared to fructose and more effectively removes glucose from ASL. Given that blood glucose concentrations are much higher than fructose in vivo, glucose rather than fructose is more important as a bacterial nutrient source and preferential transport mechanisms would therefore be physiologically relevant. Consistent with our previous study, metformin significantly raised *R*_T_ and reduced the sugar-dependent growth of *S. aureus*. Thus, epithelial paracellular permeability and glucose transport mechanisms are vital to maintaining low glucose concentration in ASL and limiting bacterial nutrient sources as a defence against infection.

### Electronic supplementary material

Below is the link to the electronic supplementary material.

Supplementary material 1 (TIFF 59 kb) Supplemental Figure 1. Sugar-induced changes in the rate of *S. aureus* growth. *S. aureus* parent strain (JE2) and mutant strains NE39(*ptsG*), NE768(*fruA*), NE172(*ptsG*), NE1944(*crr*), NE1405(*gluC*) growth in glucose-free RPMI (A), RPMI supplemented with 10 mM glucose (B), or RPMI supplemented with 10 mM fructose (C), as measured by OD_540_ over 24 h, n = 2

Supplementary material 2 (TIFF 17 kb) Supplemental Figure 2. Metformin has no significant effect on the growth of *S. aureus* strain JE2 in microbial culture. *S. aureus* (JE2) growth after 7 h in RPMI media supplemented with 10 mM glucose in microbial culture (without epithelial cells) in the presence and absence of 1 mM met form in, n = 3
