# Supplementary figures and images for: Fructose transport-deficient Staphylococcus aureus reveals important role of epithelial glucose transporters in limiting sugar-driven bacterial growth in airway surface liquid
Source: Cell Mol Life Sci. 2014 May 9;71(23):4665–73. doi: 10.1007/s00018-014-1635-y (PMC4232747; doi:10.1007/s00018-014-1635-y)

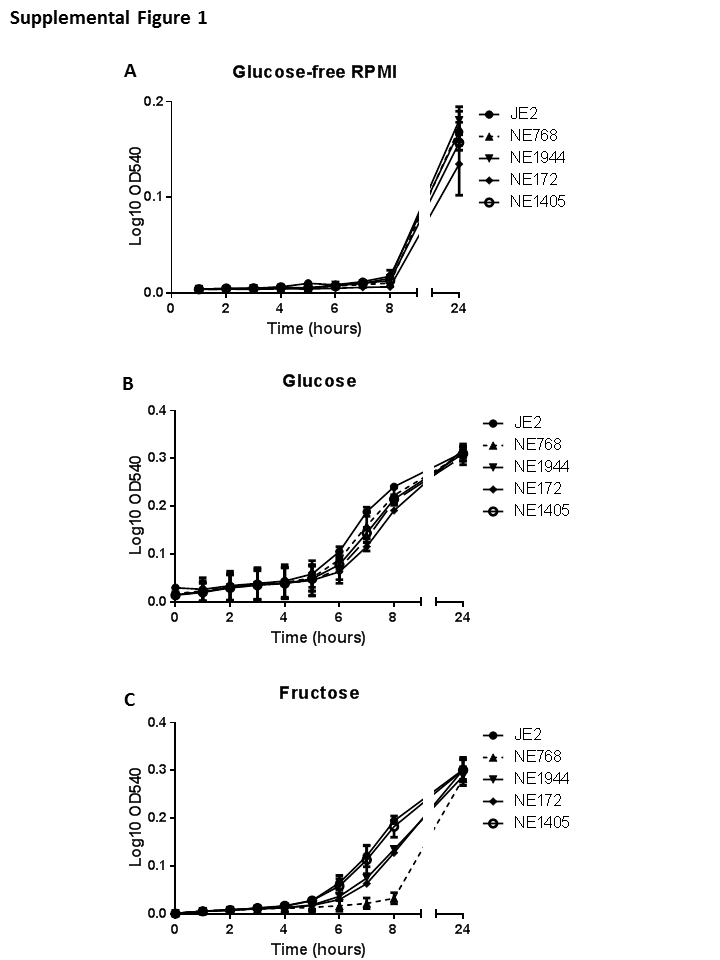

Supplement: Supplementary file 1 — Supplementary material 1 (TIFF 59 kb) Supplemental Figure 1. Sugar-induced changes in the rate of S. aureus growth. S. aureus parent strain (JE2) and mutant strains NE39(ptsG), NE768(fruA), NE172(ptsG), NE1944(crr), NE1405(gluC) growth in glucose-free RPMI (A), RPMI supplemented with 10 mM glucose (B), or RPMI supplemented with 10 mM fructose (C), as measured by OD540 over 24 h, n = 2 [file 18_2014_1635_MOESM1_ESM.tif]

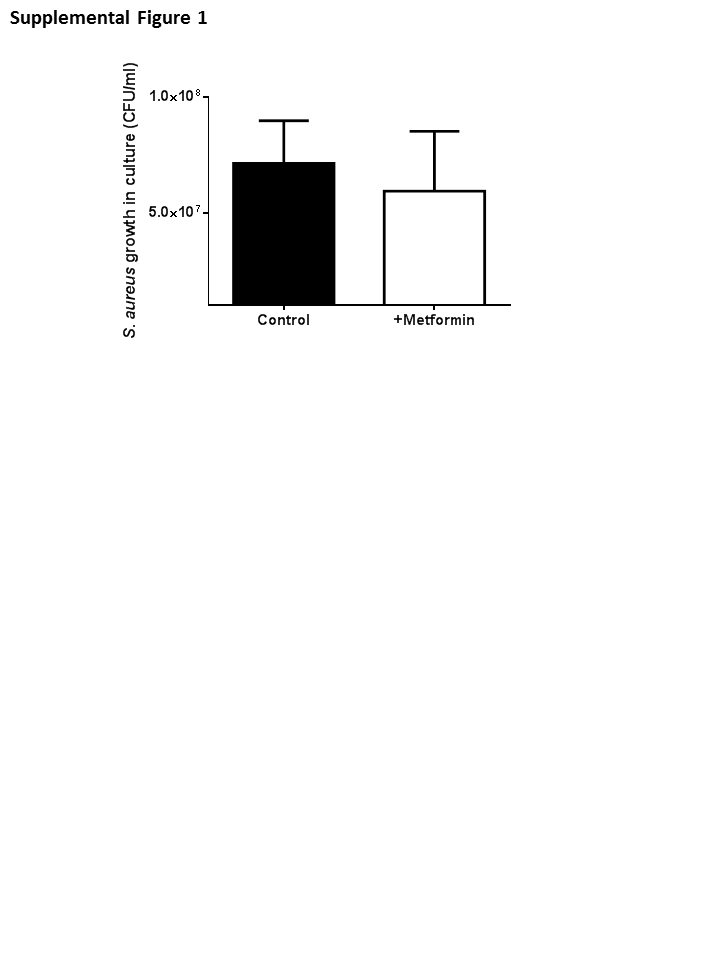

Supplement: Supplementary file 2 — Supplementary material 2 (TIFF 17 kb) Supplemental Figure 2. Metformin has no significant effect on the growth of S. aureus strain JE2 in microbial culture. S. aureus (JE2) growth after 7 h in RPMI media supplemented with 10 mM glucose in microbial culture (without epithelial cells) in the presence and absence of 1 mM met form in, n = 3 [file 18_2014_1635_MOESM2_ESM.tif]
